# Editorial: Neurovascular health insights: a powerful tool to understand and prognose neurocognitive decline

**DOI:** 10.3389/fnagi.2025.1584895

**Published:** 2025-03-14

**Authors:** Min Xia, Michael Ntim, Bin Wang

**Affiliations:** ^1^Liaoning Provincial Key Laboratory of Cerebral Diseases, Department of Physiology, College of Basic Medical Sciences, National-Local Joint Engineering Research Center for Drug Research and Development (R&D) of Neurodegenerative Diseases, Dalian Medical University, Dalian, China; ^2^Department of Anesthesiology, General Hospital of the Yangtze River Shipping, Wuhan Brain Hospital, Wuhan, China; ^3^Department of Physiology, School of Medical Sciences, College of Health Sciences, Kwame Nkrumah University of Science and Technology, Kumasi, Ghana

**Keywords:** neurocognitive decline, dementia, neurovascular health, plasma biomarkers, neuroimaging

As societies age, the global prevalence of dementia is expected to reach 139 million by 2050, with an estimated cost of $2.8 trillion by 2030 alone. However, dementia is neither an inevitable nor an unavoidable condition, up to 40% of cases can be prevented or delayed by addressing established risk factors (Long et al., 2023). Neurovascular health plays a pivotal role in the etiology, progression, and outcomes of neurocognitive decline. Impaired neurovascular coupling, characterized by reduced endothelial vasodilatory capacity (Rudnicka-Drożak et al., 2022) and diminished oxygen and glucose delivery to neurons due to decreased cerebral blood flow (Kisler et al., 2017), has been implicated in the onset and progression of dementia. Understanding the intricate interplay between vascular factors and neurodegenerative mechanisms is crucial for addressing the global burden of cognitive disorders. Advancing our understanding of neurovascular health brings us closer to mitigating cognitive decline through early detection, targeted interventions, and personalized care.

Cerebral small vessel disease (CSVD) is increasingly acknowledged as a critical contributor to neurocognitive decline, particularly in aging populations. From 2008 to 2030, nearly 5,000 publications on CSVD have appeared in 790 journals across 84 countries. Yan et al. conducted a bibliometric analysis and network visualizations of these literatures, providing insights into future research prospects. Their analysis identified magnetic resonance imaging (MRI) segmentation and enlarged perivascular spaces in the Basal ganglia as recent research interest, based on keywords co-occurrence analysis and burst graph emergence detection. MRI segmentation may enhance the diagnostic capabilities for CSVD and inspire the exploration of therapeutic targets. However, the diagnostic significance of enlarged perivascular spaces in CSVD remains unrecognized, necessitating further research into lesion differentiation and the development of quantitative methods to assess disease burden accurately.

Leveraging on its high spatial resolution, MRI uniquely visualizes subtle brain changes associated with neurocognitive decline. It is revealing that alterations in white matter integrity and cortical thinning, are crucial for understanding early-stage cognitive impairment due to vascular pathology. By integrating structural and functional imaging techniques, MRI provides a comprehensive understanding of how vascular risk factors contribute to neurocognitive decline. This facilitates the identification of biomarkers for early intervention before dementia. Zhang et al. reviewed advancements in multimode MRI, including structural MRI (sMRI), diffusion tensor imaging, resting-state fMRI, and magnetic resonance spectroscopy, in assessing vascular cognitive impairment not dementia (VCIND). They concluded that MRI not only elucidate the neurobiological mechanisms of vascular cognitive impairment (VCI) but also aids in distinguishing VCI from VCIND. Unlike MRI, Li et al. found EEG, featured by high temporal resolution, offers more precision in discriminating VCIND from healthy controls. They also demonstrated that combining EEG and sMRI using machine learning outperformed unimodal approaches in differentiating various stages of VCI. Their research highlights the possibility for early clinical diagnosis of VCI, enabling timely intervention that may delay or even reverse neurocognitive decline (Gorelick et al., 2011).

To gain deeper insight into vascular contributions to neurocognitive decline, Lim et al. examined clinical cases of post-stroke cognitive decline, focusing on brain structure, function, and metabolism. This 6-year study analyzed multimodal MRI and 18F-florbetaben PET data from 11 post-stroke cognitive decline and 10 matched non-decline controls. However, no difference was observed between the two groups on the CVSD features, including the proportion of moderate-to-severe white matter hyperintensities (WMHs) as well as the number of lacunes and microbleeds. In the decliner group, WMH volume showed a marked association with neurocognitive scores. A similar phenomenon occurred in amyloid PET characteristics, where subthreshold PET standardized uptake value ratios negatively correlated with both final neurocognitive scores and changes over time. WMH-induced white matter tract damage and amyloid pathology lead to neural network deficits that eventually trigger neurocognitive decline (Coenen et al., 2023), consistent with their study. These findings hint that neurovascular involvement in neurocognitive decline may result from multiple interacting factors.

Despite early CVSD lesions, especially before the neurocognitive decline onset, which may be evident on imaging, these changes always lag behind neurovascular lesions. Beyond imaging techniques, emerging biomarkers offer a foundation for assessing the risk and progression of neurocognitive decline. Wang Q. et al. explored the association between C677T polymorphism and neurocognitive decline in a cross-sectional study. C677T mutation is a key enzyme in homocysteine metabolism, causing high homocysteine levels, a known contributor to neurovascular injury (Hassan et al., 2004). Their study found C677T polymorphism as a genetic marker linked to elevated homocysteine levels, aggravating white matter damage and neurocognitive decline in CSVD. Likewise, Wang H. et al. explored insulin resistance as a potential mechanism underlying neurocognitive decline, highlighting its role as another contributor to neurovascular injury. They demonstrated the relationship between the triglyceride-glucose index and neurocognitive decline, emphasizing its utility as a metabolic risk indicator. Moreover, Zhao et al. confirmed baseline serum ferritin as an independent risk factor for neurocognitive decline following mild ischemic stroke and transient ischemic attack, supporting its inclusion in the predictive model of post-stroke cognitive trajectories. Their findings offer novel perspectives into understanding neurovascular health in neurocognitive decline, contributing to advancements in early detection and treatment strategies.

Collectively, these studies seek to improve patient outcomes through facilitating early diagnosis and implementing effective intervention to slow neurocognitive decline. Not just the aforementioned biomarkers, but demographics, health status, etc., also play a crucial role in determining neurocognitive decline outcomes. Wei et al. successfully developed and validated a predictive model integrating age, race, stroke, cardiovascular disease, and blood urea nitrogen to estimate 5-year mortality in neurocognitive decline patients. Their model serves as a valuable tool for prognostic evaluation and personalized care planning. Furthermore, Miranda et al. identified frailty—a state of vulnerability often linked to aging—as a mediator of poor outcomes following ischemic stroke. Their findings reveal that frailty accounted for 28% of the effect of age on disability or mortality, suggesting that mitigating frailty could enhance post-stroke recovery.

These studies highlight the significant role of neurovascular health in neurocognitive decline, emphasizing early detection and intervention. Multimodal imaging, biomarkers, and predictive models offer valuable insights into vascular contributions, aiding in personalized risk assessments. The identification of metabolic and genetic risk factors, along with the impact of frailty, underscores the need for comprehensive approaches to prevention and treatment. As dementia prevalence rises, integrating these findings into clinical practice can help delay or prevent cognitive impairment. Future research should focus on refining these diagnostic tools and intervention strategies to improve patient outcomes and quality of life.
